# Paroxysmal sympathetic hyperexcitability after brain injury: A clinical analysis of case series

**DOI:** 10.1097/MD.0000000000035375

**Published:** 2024-05-17

**Authors:** Xingru Li, Xinchen Yang, Tao Yu, Tiqiang Zhang, Yun Tang

**Affiliations:** a Department of Neurosurgery, The First Affiliated Hospital of Wannan Medical College (Yijishan Hospital of Wannan Medical College), Wuhu, China; b Department of Neurosurgery, The Second Affiliated Hospital of Nanchang University, Nanchang, China.

**Keywords:** brain injury, clinical characteristics, paroxysmal sympathetic hyperexcitability, prognosis, treatment

## Abstract

**Background:**

Paroxysmal sympathetic hyperexcitability (PSH) is a group of complex syndromes with various etiologies. Previous studies were limited to the description of traumatic brain injury (TBI), and the description of PSH after other types of brain injury was rare. We explored the clinical features, treatment, and prognosis of PSH after various types of brain injuries.

**Methods:**

Patients admitted to the neurosurgery intensive care unit with PSH after brain injury from July 2019 to December 2022 were included. Demographic data, clinical manifestations, drug therapy, and disease prognosis were retrospectively collected and analyzed.

**Results:**

Fifteen male and 9 female patients with PSH after brain injury were selected. TBI was most likely to cause PSH (66.7%), followed by spontaneous intracerebral hemorrhage (25%). Glasgow coma scale scores of 19 patients (79.2%) were lower than 8 and 14 patients (58.3%) underwent tracheotomy. Electroencephalogram monitoring was performed in 12 individuals, none of which showed epileptic waves. Clinical symptom scale showed mild symptoms in 17 cases (70.8%). Almost all patients were administered a combination of drugs. After follow-up, most patients had a poor prognosis and 2 (8.3%) died after discharge.

**Conclusion:**

The etiology of PSH is complex. TBI may be the most common cause of PSH. Non-TBI may also be an important cause of PSH. Therefore, early identification, prevention and diagnosis are helpful for determining the prognosis and outcome of the disease.

## 1. Introduction

Paroxysmal sympathetic hyperexcitability (PSH) is a group of syndromes characterized by paroxysmal agitation, hyperhidrosis, elevated blood pressure, tachycardia, tachypnea, and dystonia.^[1]^ Although the clinical features of PSH were first described in 1929,^[2]^ its name remained controversial. In previous studies,^[3,4]^ PSH was often referred to as autonomic dysfunction, autonomic storm, sympathetic storm, autonomic seizure, or hypothalamic storm. In 2010, this group of syndromes was named PSH for the first time by Rabinstein AA^[5]^ and in 2014, the expert consensus committee formally proposed the definition and diagnostic criteria for PSH.^[1]^

PSH may follow any type of acute brain injury which includes both traumatic brain injury (TBI) and non-TBI. TBI was considered an important cause of PSH and accounted for 79.4% of all causes.^[6]^ In recent years, PSH after TBI has become a research focus. Jafari A A and Perkes I E et al^[7,8]^ described the pathophysiology, clinical manifestations, duration, and management of PSH during various types of TBI. Li Z^[9]^ conducted a prospective observational study of patients with severe TBI admitted to the intensive care unit (ICU) and compared the effects of PSH on prognosis. However, non-TBI (severe intracranial infection, massive intracerebral hemorrhage, and cerebral infarction) can also be complicated with PSH, cerebral hemorrhage accounted for the largest proportion (11.3%).^[10]^ The current study focused on TBI and studies on PSH after other types of brain injuries were rare. Statistically, 7.7% to 33% of cases of PSH usually occurred in the ICU, where nonspecific symptoms of PSH can also be observed.^[11,12]^ Symptom overlap prevented PSH from being recognized and treated in a timely manner, resulting in higher mortality, longer hospital stays, higher healthcare costs, and worse disease outcomes.

Since PSH can occur after any type of brain injury, the current research were limited to TBI. This study retrospectively analyzed the clinical characteristics, treatment, and prognosis of patients with PSH after multiple types of brain damage to provide a clinical basis for future studies on PSH.

## 2. Methods

### 2.1. Study population

We studied patients with PSH after brain injury from July 2019 to December 2022. Patients with neuroleptic malignant syndrome (serotonin syndrome, autonomic nerve reflex disorder, sepsis, pulmonary embolism, and Cushing reaction) and the length of ICU stay < 14 days were excluded. Finally, 24 patients with PSH after brain injury were included in the study.

### 2.2. Data collection

Data were retrieved from electronic medical records. All patients were diagnosed with PSH using paroxysmal sympathetic hyperactivity assessment measure (PSH-AM), as recommended by the consensus.^[1]^ PSH-AM consists of clinical symptom scale (CFS) and diagnostic possibility tool (DLT). The CFS was used to evaluate the severity of PSH, the scores range from 0 to 18. The DLT was used to confirm the possibility that the 11 clinical features observed were due to PSH. PSH can be diagnosed when the total score of the CFS and DLT scores exceeded 17 points.^[1,13]^ Duration of PSH was not used as an exclusion criterion. We collected and analyzed the following demographic data and characteristics: age, sex, diagnosis, lesion site, disease course, surgical history, cerebral hernia, tracheotomy, Glasgow coma scale (GCS) score, and vital signs. Six months after discharge, the Glasgow outcome score-extended (GOS-E) was used to evaluate the prognosis and outcome of patients. This study was approved by the Ethics Committee of the First Affiliated Hospital of the Wannan Medical College (NO. YJSHLB20190601). All patients voluntarily signed informed consent.

## 3. Results

### 3.1. Characteristics of patients with PSH after brain injury

**Figure 1.**
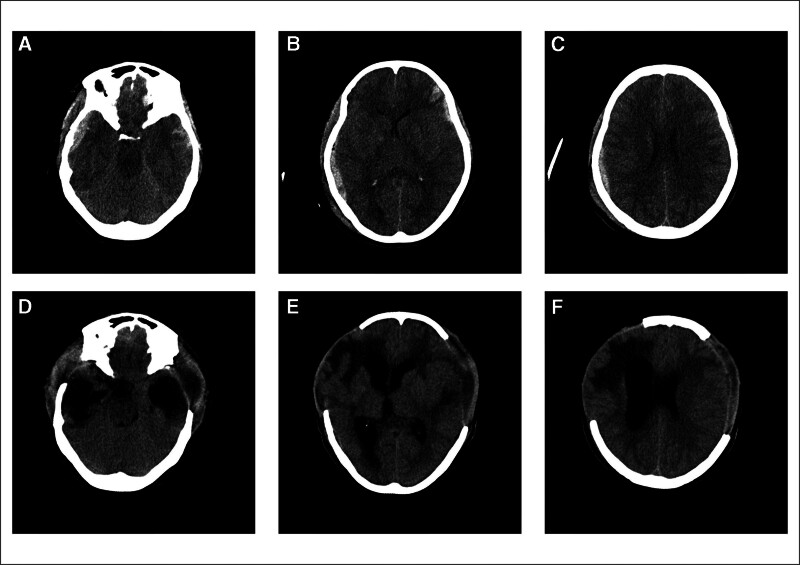
Case 1: A 41-year-old female with TBI in November 2020. Preoperative CT (A–C) showed the patient had bilateral temporal subdural hematoma with high intracranial pressure, circumferential cistern and lateral ventricle were compressed, the sulci disappeared. reexamination CT (D–F) of 10 days after surgery revealed multiple low-density lesions in the intracranial and enlarged ventricles. TBI = traumatic brain injury.

**Figure 2.**
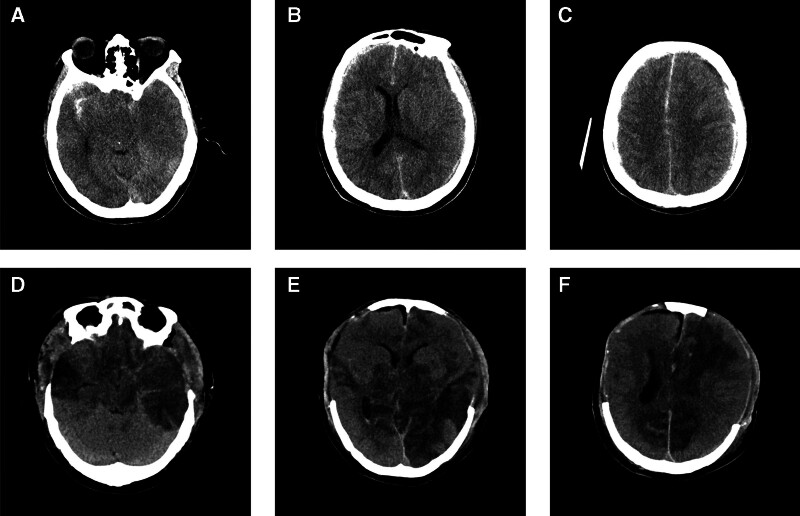
Case 1: Representative electroencephalogram (EEG) trace showed no significant epileptic waves.

**Figure 3.**
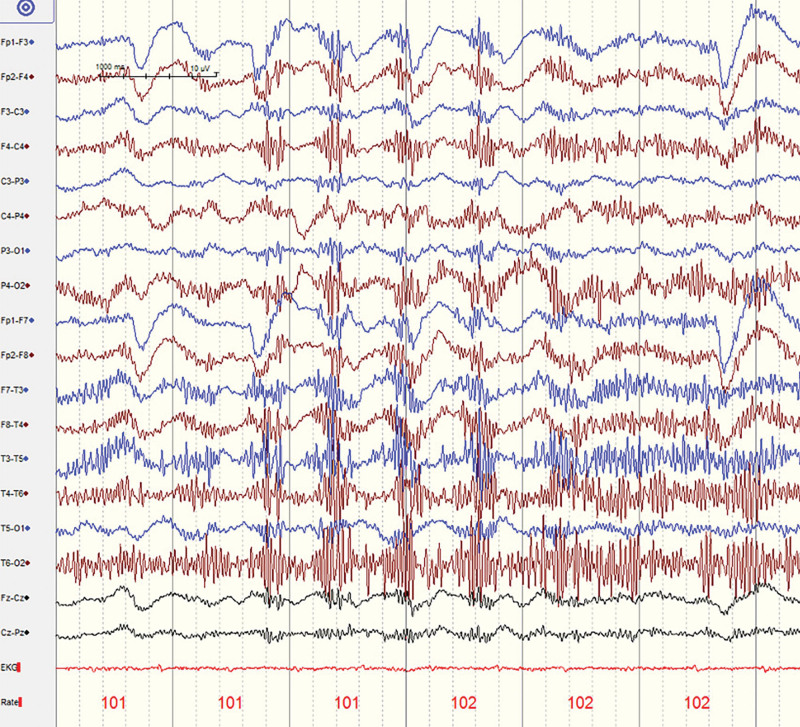
Case 2: A 50-year-old male with ICH in December 2020. Preoperative CT (A–C) showed the patient had bilateral frontotemporal parietal subdural hematoma, marked brain swelling, compression of the cisterna annulus and disappearance of cerebral sulci. reexamination CT (D–F) after surgery revealed multiple low-density changes in brain tissue. ICH = intracerebral hemorrhage.

**Figure 4.**
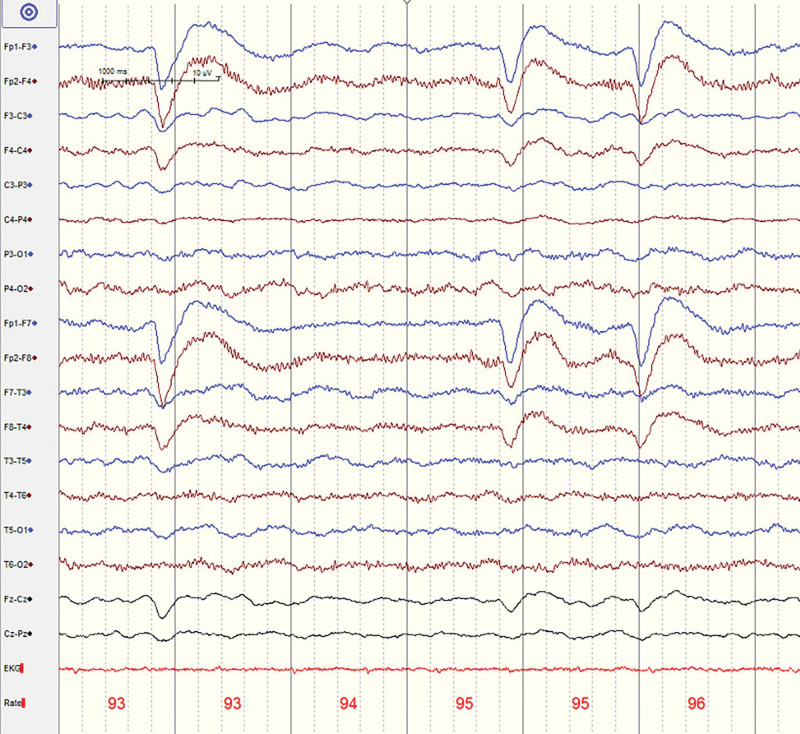
Case 2: Representative electroencephalogram (EEG) trace showed no significant epileptic waves.

A total of 24 patients were included in the study (Table 1). In this cohort, the age was 36 to 73 years (median age: 52.2 years). Fifteen patients (62.5%) were male and 9 (37.5%) were female. Four types of brain injury were included. There were 16 cases (66.7%) of craniocerebral trauma and 6 cases (25%) of spontaneous intracerebral hemorrhage (ICH), 1 case (4.2%) of cerebral infarction, and 1 case (4.2%) of brain tumor. All patients underwent computed tomography examination, brain herniation occurred in 7 patients (29.2%). Five patients (20.8%) underwent craniocerebral surgery and 3 patients (12.5%) underwent tracheotomy on admission. Tracheostomies were performed in 14 patients (58.3%) during treatment. GCS score of 19 patients (79.2%) was below 8 points. There were no obvious characteristics of admission. At the onset of PSH, the systolic pressure of 9 patients (37.5%) was exceeded 160 mm Hg, heart rate of 12 patients (50%) exceeded 120 beats per minute, respiratory rate of 17 patients (70.83%) exceeded 24 beats per minute, the temperature of 17 patients (70.83%) exceeded 38.5 °C, 11 patients (45.83%) had sweating and 9 patients (37.5%) had muscle dystonia. Electroencephalogram (EEG) monitoring was performed in 12 individuals, none of which showed epileptic waves. Furthermore, CSF scores of 17 patients (70.83%) showed 1 to 6 points, which indicated mild symptoms, 6 patients showed 7 to 12 points, which indicated moderate symptoms, and only 1 patient (4.2%) exceeded 13 points, which indicated severe symptoms. We provided CT and EEG of the first case (Figs. 1 and 2) and the second case (Figs. 3 and 4) as reference.

**Table 1:** Clinical features of patients with PSH after brain injury (n = 24).

Case	Sex/Age	Main type	Location	GCS score on admission	CSF	EGG	Drug therapy	Days of hospital stay	Days of ICU stay	GOS-E score after 6 months
1	F/41	TBI	L frontotemporal lobe	7	6	Diffuse δ and θ wave	PP, BM, BF	44	35	4
2	M/50	ICH	Bilateral frontotemporal lobes	4	4	Diffuse slow wave	MP, BM	98	31	3
3	M/51	TBI	R basal ganglia	8	12	Scattered α wave	DT, PF, MP	53	15	4
4	M/50	TBI	R thalamus	5T	4	NA	MP, PP	58	15	4
5	M/59	TBI	Lateral ventricle	3T	14	NA	PP, BF	45	19	4
6	F/49	TBI	R frontotemporal lobe	7	7	Low amplitude θ and δ wave	PP, BF, BM	39	14	3
7	F/63	TBI	R frontotemporal lobe	4	4	NA	PP, BM, BF	37	37	3
8	M/58	ICH	R thalamus	4T	6	NA	PP, BM, BF	52	35	3
9	M/63	ICH	R basal ganglia with SAH	5	4	NA	MP, BM	17	14	1
10	M/58	TBI	R basal ganglia	5T	4	Low amplitude α wave	MP	20	15	1
11	F/58	TBI	R frontotemporal lobe	10T	4	NA	PP, BM, BF	77	22	2
12	M/58	TBI	SAH	4T	8	Rhythm change	DT, PP, MP, BM, MZ	84	644	2
13	F/47	ICH	L basal ganglia	15	4	Diffuse δ and θ wave	PP	18	18	1
14	M/51	TBI	R parietal and temporal lobe with SAH	3T	6	NA	ST, PF, MZ,	55	52	2
15	F/59	ICH	L basal ganglia	6T	4	Scattered α wave	DT, ST, PF, MP, BM	70	18	3
16	F/51	TBI	L frontotemporal lobe	5T	10	NA	ST, PP, BM, BF	48	18	3
17	M/21	TBI	R frontotemporal lobe	6	8	Rhythm change	ST, DT, PF, ES	46	22	3
18	F/50	Brain tumor	Ventricles and corpus callosum	10	4	Medium amplitude θ wave	NA	28	14	4
19	M/36	TBI	L basal ganglia	4	4	NA	DT, ST, PF, MP, ES, BF, MZ	24	14	3
20	M/54	TBI	R basal ganglia	5T	4	NA	ST, PF, PP, MP BF	26	17	4
21	M/63	TBI	R basal ganglia with SAH	4T	4	Diffuse slow wave	ST, PF, BM, MZ	19	19	3
22	M/36	ICH	R basal ganglia with SAH	7T	4	Low amplitude α wave	DT, PP, ES, BM	19	19	3
23	M/54	TBI	L thalamus	9T	4	NA	ST, PP,	14	14	4
24	M/73	CI	R cerebral hemisphere	6T	11	NA	ST, PF MP	21	16	4

CI = cerebral infarction, BF = baclofen, BM = bromocriptine, CSF = clinical symptom scale, DT = dexmedetomidine, EGG = Electroencephalogram, ES = esmolol, F = female, GCS = Glasgow coma scale, GOS-E = Glasgow outcome score-extended, ICH = intracerebral hemorrhage, ICU = intensive care unit, L = left, M = man, MP = Metoprolol, MZ = midazolam, N = no, NA =not available, PF = propofol, PSH = paroxysmal sympathetic hyperexcitability, PP = Propranolol, *R* = right, SAH = subarachnoid hemorrhage, ST = Sufentanil, *T* = tracheotomy, TBI = traumatic brain injury, *Y* = yes.

### 3.2. Drug therapy and prognosis of patients with PSH after brain injury

All patients received medical treatment consisting of Propranolol, Bromocriptine, Propofol, Sufentanil, Baclofen, and Dexmedetomidine. Almost all patients received a combination of drugs and only 1 patient did not receive any drugs. Two patients received monotherapy, 19 patients were treated with fewer than 3 types of drugs, and 5 patients received more than 3 types of medication. The total length of hospital stay ranged from 14 to 98 days. Six months follow-up after discharge, The GOS-E score of the patients showed they tended to have a poor prognosis and 2 patients died.

## 4. Discussion

PSH is a rare syndrome with a complex etiology. At present, there are 2 main mechanisms of PSH: disconnection theory and the Excitation/Inhibition rate model theory. The disconnection theory suggests that the cerebral cortex and subcortical structures (upper brainstem or diencephalon) lose the inhibition of sympathetic nerves from the downstream brainstem and spinal cord pathways, resulting in sympathetic hyperexcitation. The excitation/inhibition rate model theory suggests that PSH is an imbalance between sympathetic and parasympathetic nerves that leading to increased sensitivity and activity of sympathetic nerves.^[3]^ PSH is often misdiagnosed as epilepsy, and EEG is an important way to distinguish between the 2 diseases. The clinical symptoms of PSH were not improved after antiepileptic drug treatment. The incidence of PSH after TBI is 5 times higher than that after non-TBI in the ICU.^[10]^ Our study used PSH-AM to determine PSH, although PSH-AM may reduce the possibility of misdiagnosis, it also has a high sensitivity and low specificity and may overestimate the incidence of PSH.^[14]^ Future studies will be required to clearly define the diagnostic criteria for PSH.

In addition to TBI, non-TBI was also an important cause of PSH. In our study, 25% of patients with ICH were complicated with PSH. Among these, only the fifth case had intraventricular hemorrhage which may be attributed to the fact that deep brain structural damage increased the risk of PSH.^[15]^ In our study, most patients with ICH who had bleeding on the right side developed PSH, and the most common bleeding site was the basal ganglia (37.5%), both insular cortices can influence sympathetic tone, while their effects were different. Stimulation of the left side caused bradycardia and stimulation of the right side increased heart rate, diastolic blood pressure, and sympathetic tone.^[15]^

Demographic characteristics are important factors affecting the occurrence of PSH.^[9]^ Previous study reported that men (83.3%) were more likely than women to develop PSH.^[5,16]^ Our results also showed that 62.5% of men suffered from PSH. The age distribution was relatively wide, however, it was acknowledged that younger patients had an increased risk of PSH. The age characteristics of patients with PSH were not reflected in our study due to the limited sample size. Future studies with larger sample sizes can be required to verify the age characteristics of patients with PSH.

Li^[17]^ previously reported that the GCS score of patients with PSH was significantly lower than that of patients without PSH. In our study, The GCS score of 19 patients (79.2%) below 8 on admission. It remained controversial whether the GCS score at admission was an independent risk factor for PSH. Tracheotomy was confirmed to be an independent risk factor for PSH.^[9]^ In our study, most patients did not have a history of tracheostomy on admission, however, 14 patients underwent tracheotomy during the treatment period. It remained to be explored whether tracheotomy increases the frequency of PSH. In addition, different types of patients had different periods of illness, and further studies will be needed to investigate the effects of tracheotomy in patients with different types of craniocerebral injury.

The CFS score of the patients in this study ranged from 4 to 14, and most patients had mild symptoms. In our study, the symptoms of PSH mainly manifested as increased systolic blood pressure, accelerated heart rate and breathing, which may also occur in other diseases. Therefore, it is difficult to distinguish PSH from other diseases. Early identification, prevention, and diagnosis by monitoring the indicators of patients are particularly important to reduce the incidence of PSH and decrease the severity of symptoms. In addition, changes of the vital signs may be induced by various stimuli of the external environment, such as changes in position, use of restraint bands, and suction. Therefore, nursing staff should strengthen personalized care, try to avoid stimulation, and remove inducing factors.

Early treatment with PSH can promote the recovery of the nervous system. However, the treatment plan for PSH had not been standardized and the existing evidence for treatment plans was mainly derived from clinical case reports. Currently, intravenous anesthetics, β-adrenergic blockers, α2 agonists, and benzodiazepines were used to treat patients with PSH, however, the efficacy of these drugs has not been studied. CFS score at the onset of PSH was recommended for drug prescription and dose selection to improve the accuracy of treatment. In our study, almost all the patients were treated with more than 1 drug. Bromocriptine was second only to propranolol, which was used in almost all patients. Studies has shown that single-drug intervention may not be sufficient to deal with the multiple complex clinical manifestations of PSH^[18]^ and the combined use of multiple interventions may improve prognosis. In our study, most patients were treated with a combination of these 3 types of drugs.

In our study, the length of stay in the neurosurgery intensive care unit and the total hospital stay were relatively long. A longer hospital stay was associated with poorer prognosis.^[19]^ The 6-month GOS-E scores showed most patients with PSH had poor prognosis. Two patients with cerebral hemorrhage of the basal ganglia died. Therefore, timely identification and diagnosis of PSH can shorten the length of hospital stay, reduce the cost of hospitalization, and lower the mortality rate. In addition, there were limitations in this study: this was a single-center retrospective study with a small sample size, and the assessment of clinical manifestations of PSH patients was subjective. In the future, a multi-center large-sample study can be carried out to further explore the clinical characteristics of PSH.

## 5. Conclusion

PSH, which has complex etiologies, may be secondary to different types of brain injuries. Current research was limited to PSH after TBI. Non-TBI was also a major cause of PSH. PSH can prolong the length of hospital stay for patients, increase medical costs, and seriously affect the prognosis of patients. Early identification, prevention, and diagnosis are conducive to improving disease prognosis and outcome. Future descriptive studies on the characteristics of PSH after other types of brain injury will be required to explore the risk factors, and prediction models may also be established for PSH after different types of craniocerebral injuries to lay a foundation on formulating effective prevention and standardized treatment programs.

## Acknowledgments

We thank all participants and participating departments

## Author contributions

**Conceptualization:** Tao Yu.

**Formal analysis:** Yun Tang.

**Funding acquisition:** Tao Yu.

**Investigation:** Xingru Li, Xinchen Yang, Lin Yao.

**Methodology:** Tao Yu.

**Project administration:** Tao Yu.

**Supervision:** Tao Yu, Lin Yao, Yun Tang.

**Validation:** Yun Tang.

**Writing – original draft:** Xingru Li.

**Writing – review & editing:** Tiqiang Zhang.
